# Serum midkine level and its association with subclinical coronary artery calcification and carotid atherosclerosis in chronic kidney disease

**DOI:** 10.1186/s12882-025-04066-7

**Published:** 2025-04-10

**Authors:** Osama Nady Mohamed, Marwa Ibrahim Mohamed, Shaimaa F. Kamel, Ahmed M. Dardeer, Sayed Shehata, Hassan MH Mohammed, Asmaa Khalf Kamel, Doaa Elzaeem Ismail, Nehal I. Abbas, Mohamed Ahmed Abdelsamie, Ahmed Fathy Kamel Ziady, Manar M. Sayed, Nermeen Dahi Mohammed Toni, Shaimaa Moustafa Hafez, Shereen Mohammed Mohammed Elsaghir

**Affiliations:** 1 Department of Internal Medicine, Faculty of Medicine, Minia University, Taha Hussein street, Minia, Egypt; 2 Department of Cardiology, Faculty of Medicine, Minia University, Minia, Egypt; 3 Department of Clinical Pathology, Faculty of Medicine, Minia University, Minia, Egypt; 4 Department of Radiology, Faculty of Medicine, Minia University, Minia, Egypt; 5 Department of Public and Preventive Medicine, Faculty of Medicine, Minia University, Minia, Egypt

**Keywords:** Chronic kidney disease, Atherosclerosis, Vascular calcification, Midkine

## Abstract

**Background:**

There are no studies investigating the role of midkine (MK) in vascular calcification (VC) or vascular disease associated with chronic kidney disease (CKD). This study assessed serum MK level and investigated its relationship with carotid atherosclerosis and coronary artery calcification (CAC) in non-dialysis CKD.

**Methods:**

The study comprised 80 controls and 185 adult patients with CKD at stages 3–5 who were free of cardiovascular diseases. Acute renal failure, chronic hemodialysis, severe liver disease, inflammatory states, anticoagulation therapy and cancer were excluded. The patients were classified based on presence of CAC score into severe and mild to moderate CAC groups. They were also divided into atherosclerotic and non-atherosclerotic groups based on carotid atherosclerosis. CBC, kidney function tests, lipid profile, intact parathyroid hormone (iPTH), and phosphorus were assessed. Serum levels of MK, tumor necrosis factor- α (TNF- α), interleukin-6 (IL-6), and high-sensitivity C-reactive protein (hs-CRP) were quantitatively tested using ELISA. Cardiac CT scan was done to calculate CAC score. Carotid ultrasonography was used to evaluate carotid intima media thickness (CIMT) and identify plaques.

**Results:**

All CKD categories, including CKD-3, CKD-4, and CKD-5, showed higher rates of carotid plaques (*p* = 0.007, *p* < 0.001, and *p* < 0.001, respectively), higher levels of MK (*p* < 0.001 for each), and higher CAC scores (*p* < 0.001 for each) as CKD worsened. Compared to mild to moderate CAC patients, severe CAC patients showed increased CIMT (*p* < 0.001) and raised serum levels of MK (*p* < 0.001), TNF-α (*p* = 0.001), IL-6 (*p* = 0.002), hs-CRP (*p* = 0.003), iPTH (*p* = 0.02), phosphorus (*p* < 0.001), total cholesterol (TC), and low density lipoprotein-cholesterol (LDL-C). Multivariate linear regression revealed that CAC was reliably predicted by MK (*p* = 0.008) and serum creatinine (*p* = 0.001). Carotid atherosclerotic patients had higher serum levels of MK, TNF-α, IL-6, hs-CRP, iPTH, phosphorus, TC, total triglycerides and LDL-C (*p* < 0.001 for each). Multivariate logistic regression showed that serum MK (*p* = 0.001), serum creatinine (*p* = 0.005), age (*p* < 0.001), iPTH (*p* = 0.007), and IL-6 (*p* = 0.024) were significant predictors of carotid atherosclerosis.

**Conclusions:**

As CKD worsened, MK levels, carotid atherosclerosis and CAC increased. Serum MK was a reliable biomarker for asymptomatic carotid atherosclerosis and CAC in non-dialysis CKD, allowing prompt early diagnosis to avert cardiovascular morbidity and death in the future.

**Trial registration:**

The trial number was 1138 and its registration was approved by the hospital’s Research Ethics Committee in 4/2024.

## Introduction

Chronic kidney disease (CKD) is recognized as defects in kidney function or structure that have lasted for three months or longer and have an impact on health [1]. The main risk factors for CKD include hypertension (HTN), diabetes mellitus (DM), recurrent episodes of acute kidney injury (AKI), and cardiovascular (CV) diseases (CVDs) such as heart failure. Additional risk factors comprise preeclampsia, genetics, environmental contaminants, obesity, nephrotoxic medications, and systemic lupus erythrematosus [2]. It has been proposed recently that the increased risk of atherosclerosis is actually first observed in the early stages of CKD. In addition to the traditional risk factors for atherosclerosis in CKD such as age, smoking, male gender, dyslipidemia, HTN, and physical inactivity, there are other risk factors related to CKD such as albuminuria, inflammation, vascular calcification (VC), uremic toxins, endothelial dysfunction, and oxidative stress [3].

One of the main risk factors for the development of CVD is VC, a pathological process marked by the buildup of mineral deposits on artery walls [4]. It is challenging to halt progression of coronary artery calcification (CAC) once it manifests. Therefore, it is very crucial to investigate the risk factors for VC to stop the advancement of VC from getting worse over time [5]. The existence of atheromatous disease in one or more arterial territories prior to the occurrence of any signs, symptoms, or events associated with clinically evident atherosclerotic disease in that territory is known as subclinical or asymptomatic atherosclerosis. It is a crucial early marker of atherosclerotic burden because prompt treatment could avert cardiovascular morbidity and death in the future [6]. Pre-dialysis CKD patients also have an acceleration of asymptomatic atherosclerosis, indicating that the atherosclerosis process begins prior to the initiation of renal replacement therapy. The presence of carotid plaques and CAC appear to be reliable markers of subclinical atherosclerosis [7].

Midkine (MK) is a 13 kDa cytokine which has been pathologically linked to a number of disease processes, such as cancer, acute and chronic kidney diseases and inflammatory diseases [8, 9]. MK was initially identified as a heparin binding growth factor that is highly expressed during the midgestation [10], and plays a crucial role in nephrogenesis [11]. It is expressed by proximal and distal renal tubular epithelial cells and to a lesser degree by endothelial cells [12]. It performs a variety of roles in kidney diseases, from harmful effects in acute and chronic renal diseases to promoting diabetes and HTN related injuries [13]. In CKD, MK has been implicated in endovascular, glomerular, and inflammatory tubular injuries [12]. MK is implicated as a potential trigger of the systemic diseases linked to CKD, such as vascular diseases [14], HTN [15], and cardiac dysfunction [16]. The proatherogenic impact of MK is achieved through alteration of various pathophysiological processes involved in atherogenesis, such as vascular inflammation, insulin resistance, neointima development, and macrophage lipid accumulation [17].

There are very limited human studies on MK levels in CKD. Campbell et al. examined MK levels in a population with CKD and found that MK levels in the urine and serum increased with progression of CKD, increasing by more than 10 times by CKD-5 [18]. Human researches on MK levels in peripheral atherosclerosis are scarce. Salaru et al. found that serum MK levels were considerably higher in severe PAD patients compared to healthy participants [19]. There are no precise studies investigating the role of MK in VC or vascular disease associated with non-dialysis CKD [20]. Our study was the first to measure serum MK level and to explore the association of MK with carotid atherosclerosis and CAC in non-dialysis CKD patients.

## Methods

### Study design

We conducted a cross-sectional prospective multicenter study at tertiary University centers in Minia, Egypt. The patients were selected from those seeking medical advice in outpatient clinics of our tertiary university hospitals and three central hospitals from May 2024 to October 2024. The study comprised 80 healthy individuals and 185 CKD patients. The healthy volunteers were selected from medical students and healthcare workers in our hospitals. Out of the 595 individuals who were invited, 185 patients were selected for the study and 80 subjects as a control group. 65 patients were found ineligible due to common CVDs. 45 patients declined the invitation. The patients’ refusal to participate in our study was caused by a variety of factors, including ignorance and a poor understanding of the research. Many participants, particularly those living in rural regions, were unaware of several processes used in our study. Poor timing was another factor. 37 patients had moved out of the area, and 43 did not have MK measurements available during the trial because MK kits were not available in our facilities due to financial constraints. Furthermore, some of the invited patients refused and expressed concerns because of superstition and drawing of blood. They also worried that their bodies will be used to extract more blood. Written informed consent was given by each subject. The study protocol had been approved by the hospital’s Research Ethics Committee. The Institutional Review Board’s (IRB) approval number was 1138-4-2024.

**Fig. 1:**
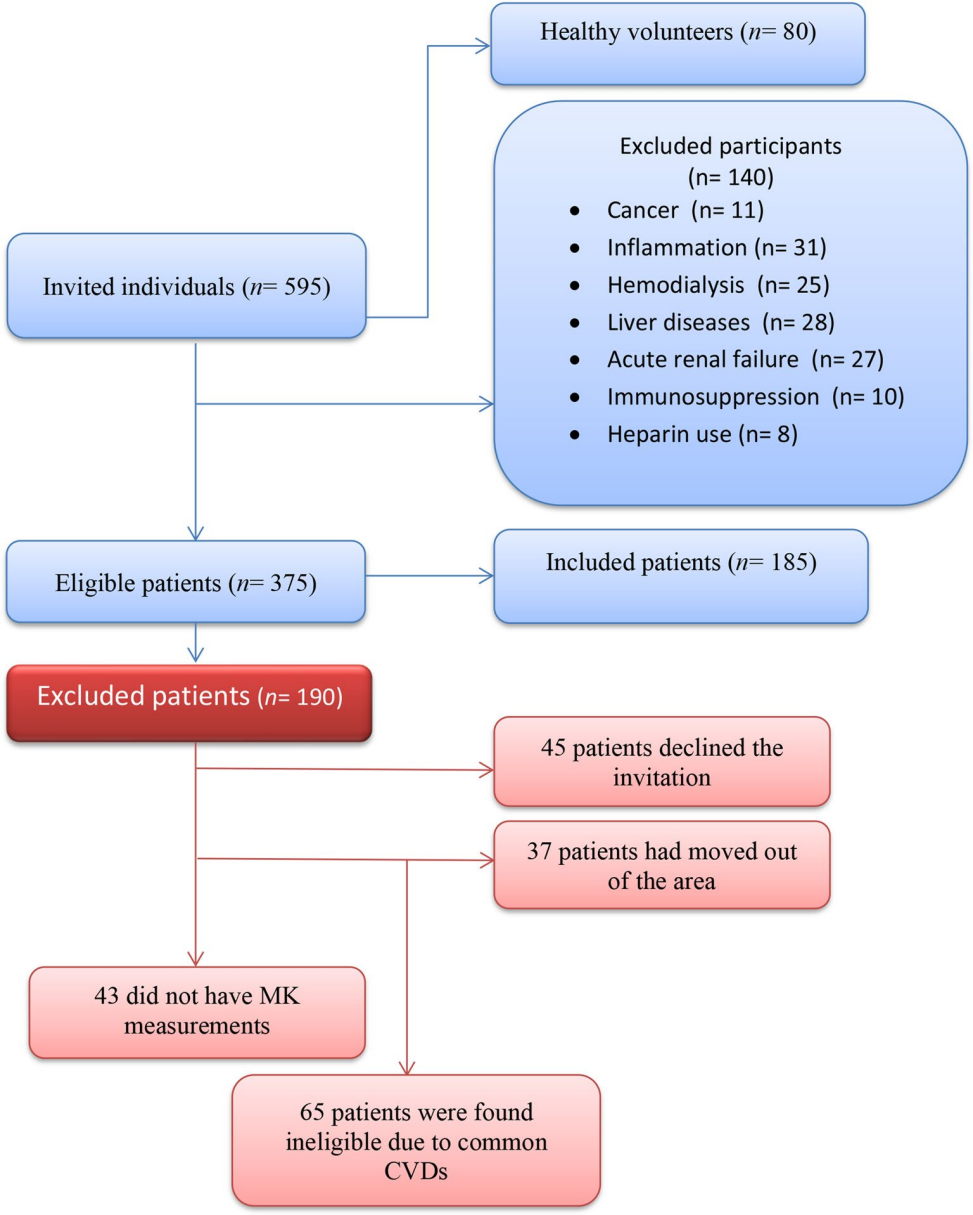
Flow-chart illustrating design of study participants

The staging and diagnosis of CKD were based on the Kidney Disease Improving Global Outcomes (KDIGO) clinical practice guidelines for evaluation and management of CKD. CKD was recognized as damage in the kidney structure or an estimated glomerular filtration rate (eGFR) of < 60 ml/min/1.73 m^2^ for more than three months. Using eGFR, the CKD patients were categorized into CKD-3 group (eGFR: 30–60 ml/min/1.73 m^2^), CKD-4 group (eGFR: 15–30 ml/min/1.73 m^2^) and CKD-5 group (eGFR < 15 ml/min/1.73 m^2^). CKD patients (stages 3–5) who were above the age of 18 years and free of common CVDs such as acute coronary syndrome (ACS), heart failure and stroke were included. Patients with acute renal failure (n = 27), patients on long-term hemodialysis (HD) (n = 25), severe liver disease (n = 28), acute or long-term inflammation (n = 31), heparin use (n = 8), immunosuppression states (n = 10) and cancer (n = 11) were excluded (Fig. 1). A comprehensive history was taken of each patient before the clinical examination. While evaluating the patient’s medical history, factors such as age, gender, smoking history, DM and HTN history, and specific CV symptoms were taken into account. The clinical examination comprised a thorough examination of the patient’s heart, belly, chest, and nervous system in addition to a detailed evaluation of the patient’s vital signs, body mass index (BMI), and peripheral vascular disease. Complete blood count (CBC), urine analysis, 24 h protein in urine, kidney function tests, lipid profile, intact parathyroid hormone (iPTH), and phosphorus were assessed. Serum levels of MK, tumor necrosis factor- α (TNF- α), interleukin-6 (IL-6), and high-sensitivity C-reactive protein (hs-CRP) were quantitatively tested using ELISA. Cardiac CT scan was done to calculate CAC score. Carotid ultrasonography was used to evaluate carotid intima media thickness (CIMT) and identify plaques.

### Sample size

Sample size formula for a qualitative variable or a quantitative variable is different. Our cross sectional prospective study was done to estimate prevalence of atherosclerotic CKD in our community. Thus, sample size formula for atherosclerotic CKD qualitative variable was calculated [21]. The formula n = (Z^2^ × P × (1-P))/d^2^ was used to get the appropriate sample size. The *Z* value reflected the desired confidence interval (CI). A 95% CI was commonly used, corresponding to a Z-score of approximately 1.96. The *P* component represented the anticipated proportion in the population based on previous studies. The *d* component was the absolute error or precision that was detected by the researchers [21]. Using this formula, a minimal sample size of 146 individuals was determined, assuming a 10.6% prevalence of CKD based on a prior study [22], with a 95% CI and a 5% absolute error/precision.

### Common cardiovascular diseases (CVDs)

CVD is one of the leading causes of death and morbidity worldwide. Globally, the number of deaths from CVD has risen by 12.5% in the last ten years [23, 24]. This increasing number of cardiovascular mortalities can be attributed to a number of factors. Ischemic heart disease (IHD) was the leading cause of the global burden of CVD in 2016, accounting for 49% of the overall burden, followed by stroke (33%). Comparatively, a considerably smaller portion of the worldwide disease burden is attributed to other CVD causes [25].

### Clinical and laboratory characteristics

A thorough history was gathered of each patient prior to a clinical examination. Age, gender, history of smoking, history of DM and HTN and detailed CV symptoms were all considered while assessing the patient’s medical history. The clinical examination included a careful assessment of the patient’s vital signs, BMI and peripheral vascular disease as well as a complete examination of the patient’s heart, abdomen, chest, and nervous system. Each patient underwent an overnight fast followed by a sterile, highly aseptic venipuncture in the morning to remove 6 ml of venous blood from a peripheral vein. Ethylene diamine tetraacetic acid (EDTA) was added to a tube containing 2 ml of blood for determining the CBC. 4 ml of blood were left for 30 minutes to clot in a simple tube. After that, the sample was centrifuged for 15 minutes at a speed of roughly 3,000 rpm. The serum was then taken and stored at − 70 °C for analysis after being expressed. This serum was used for routine laboratory tests, including those for renal functions, liver biochemistry, fasting blood glucose levels, total cholesterol (TC), low-density lipoprotein-cholesterol (LDL-C), total triglycerides (TG), high-density lipoprotein-cholesterol (HDL-C), iPTH, P and total calcium (Ca). After the measurement of fasting blood glucose, each patient had a meal containing at least 75 grams of carbohydrates. After the meal, they didn’t eat anything else for 2 h before having the test. They were instructed to rest during the two-hour waiting period because physical activity can elevate blood glucose levels. After that, 2 ml of blood was drawn to measure the blood glucose level 2 h after a meal.

Serum IL-6 was detected by enzyme linked immunoassay (EIA) using Bioassay Technology Laboratory ELISA, Changsheng S Rd, Nanhu Dist, Jiaxing, Zhejiang, China (catalog # E0090Hu). The optical density was determined at 450 nm using a microplate reader (Huma Reader 3700, Germany). Sensitivity was 1.03 pg/mL, while assay range was 2–600 pg/mL. Serum TNF-α was detected by EIA using Bioassay Technology Laboratory ELISA, Changsheng S Rd, Nanhu Dist, Jiaxing, Zhejiang, China (Cat.No # E0082Hu). The optical density was determined at 450 nm using a microplate reader (Huma Reader 3700, Germany). The standard curve range was 3–900 pg/mL. Sensitivity was 1.52 pg/mL. Serum hs-CRP was detected by EIA using ImmunoCentrix Excellence in Diagnostics and Research, Cango Park, CA 91303 USA (REF #7251042). The optical density was determined at 450 nm using a microplate reader (Huma Reader 3700, Germany). The detection range was 0.2–10 mg/L.

ELISA was utilised to identify the quantitative testing of hs-CRP, IL-6, and TNF-α. Using the Sandwich-ELISA technique, this ELISA kit was employed. The kit included a micro ELISA plate that had been pre-coated with an antibody specific to IL-6, or TNF-α. The specific antibody was applied to the micro ELISA plate wells containing standards or samples. Subsequently, each microplate well was mixed with an Avidin-Horseradish Peroxidase (HRP) combination after being successively added to a biotinylated detection antibody specific for TNF-α or IL-6. Then the incubation period ended. When the stop solution was added, the enzyme-substrate reaction was stopped, and the colour changed to yellow. The optical density (OD) was measured spectrophotometrically at a wavelength of 450 nm. Using a micro-plate reader set to 450 nm, the OD of each well was simultaneously calculated. The samples of IL-6 and TNF-a weren’t diluted with this kit. Owing to the material we use to prepare the kit; the sample matrix interference may falsely depress the specificity and accuracy of the assay.

ELISA test operating in solid phase was used to develop the hs-CRP ELISA. An exclusive monoclonal antibody was used in the assay procedure to target a specific antigenic determinant on the CRP molecule. Absorbance was measured spectrophotometrically at 450 nm. In 15 minutes, the absorbance at 450 nm was read using the microtiter reader. The patient samples and controls were diluted 1:100 by adding 5 µl of samples to 495 µl of sample diluent.

Human MK assay was determined by EIA using Bioassay Technology Laboratory ELISA, Changsheng S Rd, Nanhu Dist, Jiaxing, Zhejiang, China (Cat.No #E1633Hu). The optical density was determined at 450 nm using a microplate reader (Huma Reader 3700, Germany). The standard curve range was 5–2000 pg/mL. Sensitivity was 2.49 pg/mL. The sandwich ELISA method was the foundation of this kit. The well plate had been pre-coated with an antibody. The wells were filled with standards, test samples, and biotin-conjugated reagent before being incubated. After adding the HRP-conjugated reagent, the plate was incubated as a whole. Wash buffer was used at each stage to eliminate unbound conjugates. Quantification of the HRP enzymatic reaction was done using TMB substrate. Only wells with enough MK yielded a blue product once the TMB substrate was supplied; this product became yellow when the acidic stop solution was applied. The amount of MK bound on the plate was proportionate to the intensity of the yellow colour. The concentration of MK was estimated by measuring OD spectrophotometrically in a microplate reader at 450 nm. The sample wasn’t be diluted with this kit. Owing to the material we use to prepare the kit; the sample matrix interference may falsely depress the specificity and accuracy of the assay.

### Imaging investigations

12 Lead resting surface ECG and echocardiography were done to identify people who had ischemic heart disease (IHD) or heart failure. Comprehensive two- and three-dimensional transthoracic echocardiography was aimed to evaluate abnormalities in the resting segmental wall motion, measure the left ventricular ejection fraction precisely using the Simpson biplane method and M-mode, assess diastolic function based on tissue Doppler and diastolic mitral flow Doppler, measure the dimensions and volumes of the left ventricle during both systole and diastole, and assess ischemic mitral regurgitation [26].

**Fig. 2:**
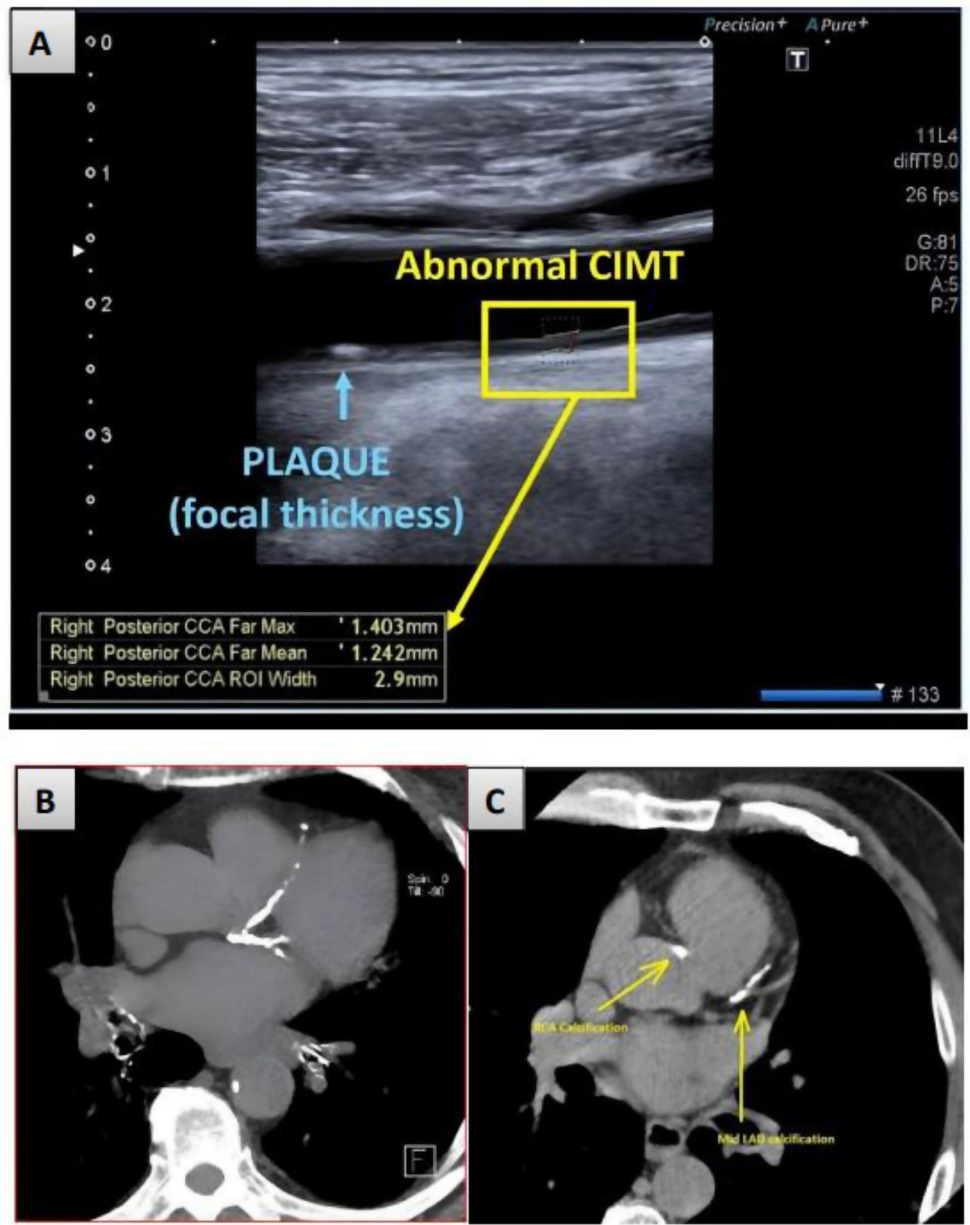
Carotid duplex and cardiac CT scan in the studied patients: (**A**) Carotid duplex showing increased carotid intima media thickness (CIMT) and carotid atheromatous plaque; (**B**) Cardiac CT demonstrating heavily calcified coronary vessels with a score over 2000; (**C**) Cardiac CT demonstrating coronary calcification in left anterior descending coronary artery (LAD) and right coronary artery (RCA)

After drawing blood samples for MK measurements, carotid ultrasound and cardiac spiral computed tomography (CT) were conducted at a follow-up session (Fig. 2). In order to calculate CAC score, cardiac spiral CT was utilised [27]. Every study participant underwent bilateral carotid examination utilising Xario-200 Toshiba ultrasonography (Toshiba, Tokyo, Japan). The patients were lying supine or semi-supine, with their head rotated 45 degrees away from their sides and slightly extended. Higher-frequency linear transducers (12 MHz) were used to measure the carotid intima media thickness (CIMT) and to evaluate the morphology of the plaque shape. Linear transducers working at a lower frequency of 7 MHz were preferred for Doppler investigations. Gray-scale imaging was used to evaluate the location, size, and features of the atherosclerotic plaque in the common carotid artery (CCA) and internal carotid artery (ICA). Regions of aberrant blood flow that needed Doppler spectrum analysis were identified using colour Doppler imaging. Assessing the velocity of blood flow in the mid-CCA, proximal ICA, and near, at, and immediately distal of the afflicted areas required performing a pulsed wave Doppler spectrum analysis. Plaque had been assessed and classified as heterogeneous, homogeneous, smooth, or irregular. The far or near walls of bulb, CCA, and ICA were used for determining the CIMT. Only the intima (echo-genic layer) and the media (echo-poor layer) were affected. Any of the following was thought to be compatible with the arteriosclerosis diagnostic criteria: (1) CIMT > 1 mm; (2) Carotid stenosis or plaque [28].

The cardiac CT scan was conducted by a qualified radiologist using a Toshiba 640-slice CT scanner (Toshiba, Tokyo, Japan). CAC was scored using the Agatston scoring method, which was provided by the KDIGO. Lesion area (mm2) multiplied by a density factor yielded the lesion score [29]. The weighted density scores were graded as follows: 130–199 hounsfield units (HU): 1, 200–299 HU: 2, 300–399 HU: 3, and ≥ 400 HU: 4. The scores for each lesion were added to get the overall CAC score. A score of 0 represented no risk of coronary calcification, while scores of 1–100, 101–400, and > 400 indicated mild, moderate, and severe risk, respectively [30].

The intimal and medial VCs constitute discrete clinical entities with unique pathogenic signatures and it appears that the fundamental pathogenic principles are still relevant in the CKD subjects (intimal calcification - atherosclerosis and medial calcification - loss of the cushioning function). Specifically, both forms of VC share oxidative and inflammatory signaling pathways, which are significantly triggered by uremic circumstances [31]. Accordingly, intimal and medial VC in CKD patients can often occur simultaneously, and multiple studies have documented the co-occurrence of the two VC forms [32].

Large superficial vessels, including the carotid and femoral arteries, can be evaluated with ultrasound [33]. According to earlier research, calcified carotid plaques found by ultrasonography were an independent risk factor for cardiovascular events in individuals with CKD [34] and were substantially correlated with the conventional risk factors of CVD [35]. There are subjective and operator-dependent limits to ultrasound and it can be challenging to distinguish between medial and intimal calcifications. Ultrasound, on the other hand, may be a practical substitute for VC assessment since it can evaluate the pulse wave velocity, which is a measure of arterial compliance and stiffness, and inspect each vessel in detail without exposing users to radiation [36].

Compared to other imaging modalities, CT is more objective and sensitive for VC evaluation. Numerous studies have demonstrated that the CT measurement of VC has a good predictive value for cardiovascular events and excellent sensitivity and specificity [37]. CT-detected coronary artery and aortic calcification has a well-established clinical importance. A scoring method to measure CAC was proposed by Agatston et al. and has a good sensitivity and specificity for predicting cardiovascular events [38]. Additionally, CT offers the benefit of monitoring the development of VC over time and acquiring extra data including bone density and valvular calcification [39]. The disadvantages of CT include its high radiation exposure risk, high expense, and limited accessibility [40]. Moreover, its application for risk classification in CKD patients is restricted due to the challenge of differentiating between intimal and medial calcification. Several researches have proposed employing a combination of biomarkers and other risk variables for reducing this restriction. This field requires more studies [41, 42].

### Study covariates

Coronary calcification and carotid atherosclerosis were the dependent outcome variables, and were adjusted for independent variables including age, smoking, DM, HTN, hs-CRP, IL-6, serum TG, LDL-C, iPTH, phosphorus, and MK. A series of univariate logistic regression analyses were performed to compute the odds ratio (OR) and 95% confidence interval (CI) for carotid atherosclerosis adjusted for the potential risk factors including age, smoking, DM, HTN, high hs-CRP, IL-6, TG, LDL-C, iPTH, phosphorus, and MK. Similarly, the Beta value and 95% CI for coronary calcification, adjusted for potential risk factors such as age, smoking, DM, HTN, high hs-CRP, IL-6, serum triglycerides, LDL-C, iPTH, phosphorus, and MK, were also calculated using a series of univariate linear regression analyses. Similarly, the Beta value and 95% CI for MK levels, adjusted for potential risk factors such as age, DM, proteinuria, medications use including ACEI, ARBs, CCBs, BB, insulin, and oral hypoglycemic drugs, high hs-CRP, IL-6, serum triglycerides, HDL-C, LDL-C, iPTH, and phosphorus, were also calculated using a series of univariate linear regression analyses. Variables that showed an association with coronary calcification, carotid atherosclerosis, or serum MK level at a significant level of *P* < 0.05 during univariate regression were included in the multivariate regression.

### Statistical analysis

SPSS version 25 was employed for data analysis. Categorical data were displayed using numbers and percentages, while continuous data were displayed using medians and interquartile ranges (IQR) or means with standard deviations (SD). The Mann Whitney and Kruskal Wallis tests were used to assess non-parametric continuous variables in comparisons between two or more groups, respectively. To analyse categorical data, the chi-squared test was used. The Pearson correlation coefficient was utilised to examine the association between two continuous variables, whereas the Spearman correlation coefficient was employed to determine the relationship between continuous and categorical variables or two categorical variables. The receiver operating characteristic (ROC) curve was created using Medcalc statistical analysis. Youden index was used to evaluate the cut-off value, the specificity, the sensitivity, negative predictive value (NPV), positive predictive value (PPV), and the area under the curve (AUC) of serum MK to predict carotid atherosclerosis and CAC. Predictors of carotid atherosclerosis were identified by the use of logistic regression analysis. Univariate and multivariate linear regression analyses were utilised to identify the predictors of CAC. Additionally, linear regression was utilised to determine variables affecting serum MK. Regarding regression analysis utilised to identify predictors of MK, CAC, and carotid atherosclerosis, we utilised the variables that were found to be significantly correlated with MK, CAC, and carotid atherosclerosis because establishing correlation is a prerequisite for regression. We didn’t use regression unless there was a significant correlation. Moreover, the variance inflation factor (VIF) and tolerance were used to statistically measure the multicollinearity of regression analysis. Multicollinearity was present if the VIF was greater than 5 to 10 and the tolerance was less than 0.1 to 0.2.

## Results

### Serum MK level and subclinical CVDs in the studied CKD subgroups

As CKD deteriorated, all patients in CKD subgroups, including CKD stage-3, CKD stage-4 and CKD stage-5 had statistically higher levels of MK than the controls (*p* < 0.001 for each). Additionally, it was discovered to be substantially greater in CKD stage-5 and CKD stage-4 patients than in CKD stage-3 patients (*p* < 0.001 for each). There was a significant increase in CIMT of CKD stage-3, CKD stage-4 and CKD stage-5 subgroups compared to the normal subjects (*p* = 0.004, *p* < 0.001, and *p* < 0.001, respectively). CIMT was also considerably increased in CKD stage-5 subgroup compared to CKD stage-3 subgroup (*p* < 0.001). CKD stage-3, CKD stage-4 and CKD stage-5 subgroups had higher rates of carotid plaques when compared to the healthy subjects (*p* = 0.007, *p* < 0.001, and *p* < 0.001, respectively). Furthermore, CKD stage-5 patients had a significantly higher level of plaques compared to CKD stage-3 patients (*p* = 0.03). We also discovered that CAC was found to be significantly increased in CKD stage-3, CKD stage-4 and CKD stage-5 subgroups compared to the healthy individuals (*p* < 0.001 for each). Furthermore, we observed that CKD stage-3, CKD stage-4 and CKD stage-5 groups had significantly higher CAC scores than the healthy individuals (*p* < 0.001 for each). Additionally, CAC scores of CKD stage-5 patients were greater than those of CKD stage-3 patients (*p* < 0.001) (Table 1).

**Table 1:** MK level and subclinical CVDs in CKD subgroups

Variables	Group I (Controls) n = 80	Group II (CKD– 3) n = 61	Group III (CKD– 4) n = 62	Group IV (CKD– 5) n = 62	I vs II	I vs III	I vs IV	II vs III	II vs IV
					*p* value				
MK (pg/mL)	700 (310) 390–890	1400 (375) 1150–2400	2760 (2345) 1080–4850	3400 (4375) 1350–9800	< 0.001	< 0.001	< 0.001	< 0.001	< 0.001
Carotid atherosclerosis									
1. CIMT (mm)	0.6 (0.07)	0.7 (0.25)	0.85 (0.8)	0.9 (0.7)	0.004	< 0.001	< 0.001	0.29	< 0.001
2. Plaques (%)									
•Positive	0 (0%)	13 (21.3%)	20 (32.3%)	24 (38.7%)					
•Negative	40 (100%)	48 (78.7%)	42 (67.7%)	38 (61.3%)	0.007	< 0.001	< 0.001	0.17	0.03
CAC score	0.0 (0.0)	240 (290)	295 (412.5)	670 (842.5)	< 0.001	< 0.001	< 0.001	0.62	< 0.001
•No CAC	40 (100%)	20(32.8%)	20(32.3%)	16 (25.8%)					
•Mild to moderate	0.0 (0.0%)	41(67.2%)	22(35.5%)	7 (11.3%)					
CAC									
•Severe CAC	0.0 (0.0%)	0 (0%)	20(32.3%)	39 (62.9%)					

CKD, chronic kidney disease; CIMT, carotid intima media thickness; CAC, coronary artery calcification; MK, midkine

Continuous variables were expressed as median (interquartile range), while categorical variables were expressed as number and percentage

### Predictors of serum MK in the CKD patients

Age, serum creatinine, hs-CRP, and IL-6 were found to be independently associated with serum MK (*p* < 0.001 for each) according to univariate regression. Additionally, it reported that TG, LDL-C, HDL-C, iPTH and phosphorus were significant predictors of MK level (*p* < 0.001 for each). Regarding medications use, we found that use of angiotensin blockade (ACEI: angiotensin converting enzyme inhibitor, or ARBs: angiotensin receptor blockers) was negatively associated with serum MK (*p* < 0.02). Insulin and oral hypoglycemic drugs didn’t significantly affect serum MK. Additionally, serum MK was not linked to diabetes mellitus, although it was favorably associated with proteinuria (*p* < 0.001) (Table 2). Multivariate linear regression revealed that age, and serum creatinine were independently associated with serum MK (*p* = 0.007, and *p* < 0.001, respectively). Additionally, it showed that TG and HDL-C emerged as statistically significant independent factors influencing serum MK (*p* < 0.001, and *p* = 0.006, respectively). Moreover, multivariate linear regression showed that use of angiotensin blockade was negatively associated with serum MK (*p* < 0.02) (Table 3).

**Table 2:** Univariate linear regression to determine predictors of MK level in the CKD patients

Variables	Univariate linear regression						
	Unstandardized Coefficients		Standardized Coefficients	t	*p* value	Confidence interval (CI)	
	B	SE	Beta			Lower bound	Upper bound
Age	118.90	17.24	0.45	6.89	< 0.001	84.89	152.92
DM	339.76	308.78	0.08	1.10	0.27	−269.46	948.97
Proteinuria	1455.56	259.47	0.35	5.61	< 0.001	944.23	1966.89
Medications							
ACEI/ARBs	−704.53	293.72	−.16	−2.39	0.02	−1283.35	−125.71
CCBs/BB	1466.94	393.91	0.24	3.72	< 0.001	690.67	2243.21
Insulin	357.69	379.06	0.06	0.94	0.34	−389.31	1104.69
Oral hypoglycemics	451.24	347.81	0.09	1.29	0.20	−234.17	1136.67
Creatinine	884.42	69.53	0.68	12.72	< 0.001	747.23	1021.60
IL-6	70.13	6.17	0.65	11.37	< 0.001	57.92	81.73
hs-CRP	46.37	5.54	0.53	8.37	< 0.001	35.44	57.30
TG	37.74	2.76	0.71	13.66	< 0.001	32.29	43.19
HDL-C	−110.41	29.89	−0.26	−3.69	< 0.001	−169.39	−51.44
LDL-C	49.02	6.17	0.51	7.94	< 0.001	36.84	61.19
iPTH	4.85	1.21	0.28	4.01	< 0.001	2.46	7.24
P	993.79	217.14	0.32	4.57	< 0.001	565.37	1422.21

ACEI, Angiotensin converting enzyme inhibitor; ARBs, Angiotensin receptor blockers; CCBs, Calcium channel blockers; BB, B-blocker; IL-6, interleukin-6; hs-CRP, high sensitivity C-reactive protein; TG, total triglyceride; LDL-C, low density lipoprotein-cholesterol; HDL-C, high density lipoprotein-cholesterol; iPTH, intact parathyroid hormone; P, phosphorus; MK, midkine; SE, standard error; CI, confidence interval

**Table 3:** Multivariate linear regression to determine predictors of MK level in the CKD patients

Variables	Multivariate linear regression						
	Unstandardized Coefficients		Standardized Coefficients	t	*p* value	Collinearity Statistics	
	B	SE	Beta			Tolerance	VIF
Age	33.99	12.40	0.130	2.74	0.007	0.59	1.70
Proteinuria	277.13	169.90	0.068	1.63	0.105	0.75	1.33
ACEI/ARBs	−424.69	185.57	−0.097	−2.29	0.02	0.74	1.36
Creatinine	564.81	62.16	0.437	9.09	< 0.001	0.57	1.76
IL-6	8.65	6.22	0.081	1.39	0.17	0.39	2.54
hs-CRP	6.61	4.31	0.075	1.53	0.12	0.55	1.82
TG	19.49	3.21	0.367	6.07	< 0.001	0.36	2.77
HDL-C	−57.85	20.78	−0.138	−2.78	0.006	0.54	1.87
LDL-C	−2.51	5.32	−0.026	−0.47	0.64	0.44	2.29
iPTH	−0.08	0.76	−0.005	−0.10	0.92	0.66	1.52
P	28.29	135.86	0.009	0.21	0.83	0.69	1.46

ACEI; Angiotensin converting enzyme inhibitor, IL-6, interleukin-6; hs-CRP, high sensitivity C-reactive protein; TG, total triglyceride; LDL-C, low density lipoprotein-cholesterol; HDL-C, high density lipoprotein-cholesterol; iPTH, intact parathyroid hormone; P, phosphorus; MK, midkine; SE, standard error; VIF, variance inflation factor

### Demographic and laboratory characteristics of carotid atherosclerotic and non-atherosclerotic groups

We then divided the CKD patients into carotid atherosclerotic and non-atherosclerotic groups according to the existence of carotid atherosclerosis. The median age of atherosclerotic patients who included 36 males and 24 females, was 49 years. Non-atherosclerotic patients who consisted of 67 males and 58 females had a median age of 43 years. The atherosclerotic patients were older than non-atherosclerotic patients (*p* < 0.001). The atherosclerotic patients had considerable increase of HTN, DM and smoking compared to non-atherosclerotic patients (*p* < 0.001, *p* < 0.001, and *p* = 0.001, respectively). The atherosclerotic group had a considerably lower eGFR (*p* = 0.017) and a significantly greater serum level of creatinine (*p* < 0.001) compared to the non-atherosclerotic group. Atherosclerotic patients demonstrated significantly elevated serum levels of TNF-α, IL-6, hs-CRP, TC, TG, LDL-C, iPTH and phosphorus (*p* < 0.001 for each) in comparison to the non-atherosclerotic patients. Furthermore, we observed that the atherosclerotic patients had a considerably higher serum MK level (*p* < 0.001), a higher CAC score (*p* < 0.001) and a significantly increase of CIMT (*p* < 0.001) in comparison to non-atherosclerotic patients (Table 4).

**Table 4:** Demographic and laboratory parameters of carotid atherosclerotic and non-atherosclerotic groups

Variables	Carotid atherosclerotic group n = 60	Carotid non-atherosclerotic group n = 125	*p* value
Age (yr)	49 (9)	43 (10)	< 0.001
Sex, *n* (%)			
Male	36 (60%)	67 (53.6%)	0.41
Female	24 (40%)	58 (46.4%)	
HTN, *n* (%)	34 (56.7%)	33 (26.4%)	< 0.001
DM, *n* (%)	38 (63.3%)	31 (24.8%)	< 0.001
Smoking, *n* (%)	29 (48.3%)	30 (24%)	0.001
BMI (kg/m2)	22.1 (1.0)	22.4 (1.12)	0.97
Serum creatinine (mg/dL)	4.25 (2.5)	2.7 (2.35)	< 0.001
Blood urea (mg/dL)	75 (21)	69 (23)	0.054
eGFR (ml/min/1.73 m2)	22 (15)	31 (20.75)	0.017
Hb (gm/dL)	9.85 (2.9)	10 (1.5)	0.34
TNF-α (pg/mL)	40.25 (26)	14.6 (11)	< 0.001
IL-6 (pg/mL)	38 (40.5)	14.9 (12.1)	< 0.001
hs-CRP (mg/L)	48 (34.5)	30 (12)	< 0.001
TC (mg/dL)	210 (32.5)	180 (25)	< 0.001
TG (mg/dL)	220 (81.25)	160 (20)	< 0.001
LDL-C(mg/dL)	128 (31)	109 (19.5)	< 0.001
HDL-C (mg/dL)	35 (5)	40 (10)	< 0.001
iPTH (pg/mL)	372.5 (140)	154 (135)	< 0.001
P (mg/dL)	6.3 (1.22)	6 (0.7)	< 0.001
Total Ca (mg/dL)	7.8 (0.63)	8 (0.3)	< 0.001
CIMT (mm)	1.4 (0.12)	0.7 (0.2)	< 0.001
MK (pg/mL)	4000 (3660)	1650 (1225)	< 0.001
CAC score (HU)	430 (568.5)	265 (370)	< 0.001

Hb, hemoglobin; HTN, hypertension; BMI, body mass index; TNF-α, tumor necrosis factor- α; IL-6, interleukin-6; hs-CRP, high sensitivity C-reactive protein; TC, total cholesterol; TG, total triglyceride; HDL-C, high density lipoprotein-cholesterol; LDL-C, low density lipoprotein-cholesterol; iPTH, intact parathyroid hormone; P, phosphorus; Ca, calcium; CIMT, carotid intima media thickness; MK, midkine; CAC, coronary artery calcification; HU, hounsfield units. Continuous variables were expressed as median (interquartile range), while categorical variables were expressed as number and percentage

### Predictors of carotid atherosclerosis in the CKD patients

**Fig. 3:**
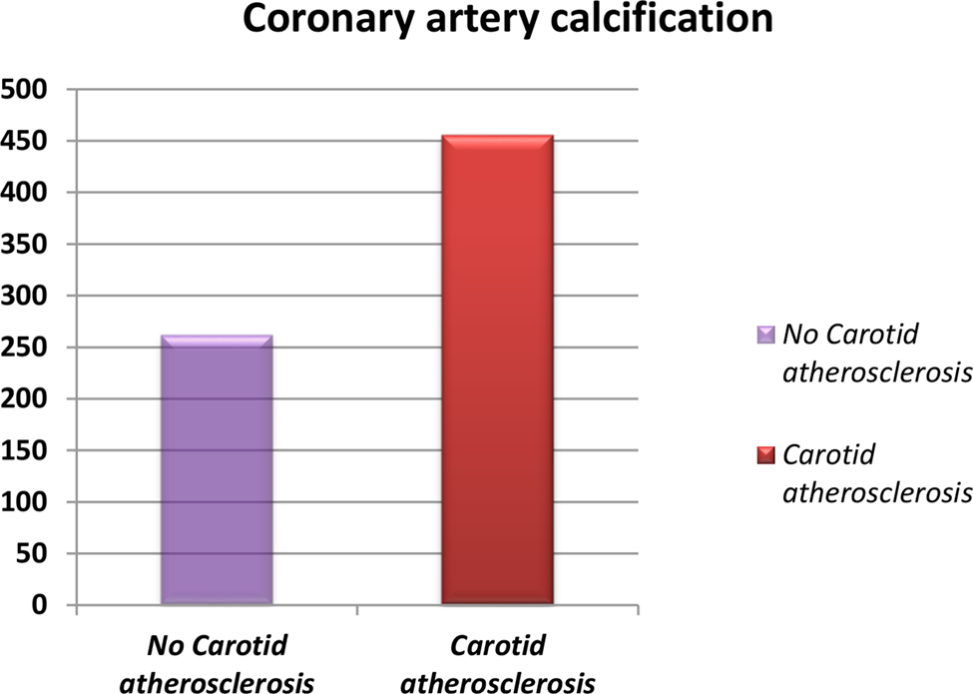
Bar chart showing that coronary artery calcification (CAC) was considerably increased in carotid atherosclerotic patients compared to non-carotid atherosclerotic patients. Furthermore, CAC was positively correlated with carotid atherosclerosis (r = 0.29, *P* < 0.001)

**Fig. 4:**
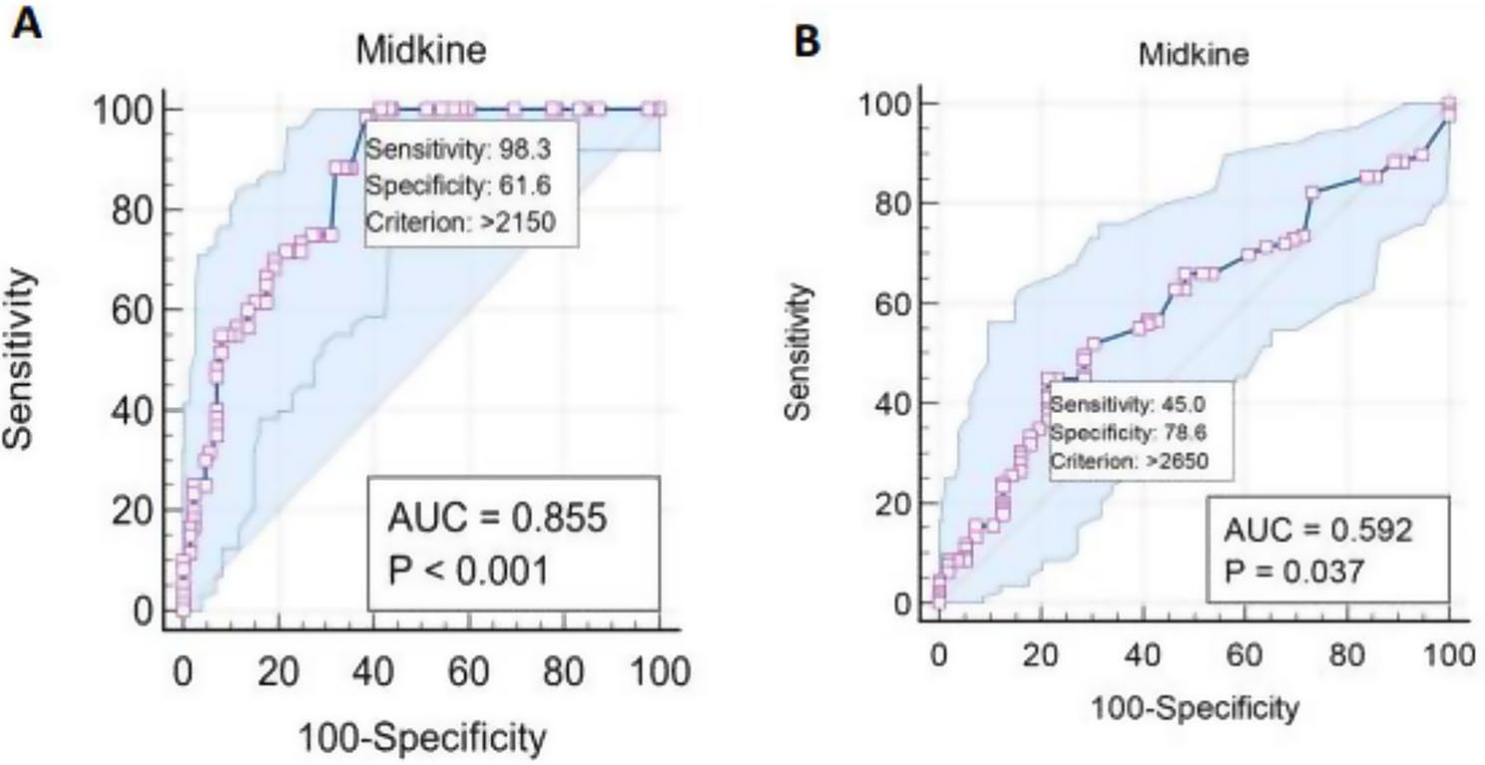
Receiver operating characteristic curve (ROC) showing that serum midkine (MK) is a predictor of carotid atherosclerosis and coronary artery calcification (CAC): (**A**) ROC curve showing accuracy of MK in predicting carotid atherosclerosis. Serum MK is a reliable predictor of subclinical carotid atherosclerosis (AUC [Area under the curve] = 0.855, *p* < 0.001); (**B**) ROC curve showing accuracy of serum MK in predicting CAC. CAC is reliably predicted by serum MK (AUC = 0.592, *p* < 0.037)

Univariate regression demonstrated that serum creatinine (odds ratio [OR]: 1.5, *p* < 0.001), age (OR: 1.29, *p* < 0.001), smoking (OR: 2.96, *p* = 0.001), DM (OR: 5.24, *p* < 0.001) and HTN (OR: 3.65, *p* < 0.001) were considerably associated with carotid atherosclerosis. TG (OR: 1.02, *p* < 0.001), LDL-C (OR: 1.04, *p* < 0.001), iPTH (OR: 1.01, *p* < 0.001), and P (OR: 3.28, *p* < 0.001) were independently associated with carotid atherosclerosis. It also found that higher serum levels of MK (OR: 1.01, *p* < 0.001), hs-CRP (OR: 1.03, *p* < 0.001) and IL-6 (OR: 1.06, *p* < 0.001) were significant predictive factors for carotid atherosclerosis. CAC was positively correlated with carotid atherosclerosis (r = 0.29, *p* < 0.001) (Fig. 3). Multivariate regression found that serum MK (OR: 1.001, *p* = 0.001), serum creatinine (*p* = 0.005) and age (OR: 1.26, *p* < 0.001) were reliable predictive risk factors of carotid atherosclerosis. It also reported that iPTH (OR: 1.01, *p* = 0.007), and IL-6 (OR: 1.06, *p* = 0.024) were significant predictors for advancement of carotid atherosclerosis (Table 5). ROC curve demonstrated that MK was a significant biomarker for carotid atherosclerosis (AUC = 0.855, *p* < 0.001) (Fig. 4). Age (AUC = 0.868, *p* < 0.001), creatinine (AUC = 0.685, *p* < 0.001), IL-6 (AUC = 0.767, *p* < 0.001), hs-CRP (AUC = 0.774, *p* < 0.001), TG (AUC = 0.690, *p* < 0.001), and LDL-C (AUC = 0.748, *p* < 0.001), were all reliable predictors of carotid atherosclerosis according to ROC curve analyses. Moreover, iPTH (AUC = 0.764, *p* < 0.001), P (AUC = 0.671, *p* < 0.001), and MK (AUC = 0.855, *p* < 0.001) had the accuracy to predict carotid atherosclerosis in the studied patients (Table 6).

**Table 5:** Logistic regression analyses to determine predictors of carotid atherosclerosis in the studied patients

Variables	Univariate logistic regression				Multivariate logistic regression			
	*P* value	Exp (B)	95% CI		*P* value	Exp (B)	95% CI	
			Lower bound	Upperbound			LowerBound	Upper bound
Age	< 0.001	1.29	1.19	1.40	< 0.001	1.26	1.11	1.43
Smoking	0.001	2.96	1.54	5.68	0.24	2.21	0.59	8.19
DM	< 0.001	5.24	2.69	10.17	0.15	3.17	0.66	15.19
HTN	< 0.001	3.65	1.91	6.96	0.48	1.66	0.41	6.74
Creatinine	< 0.001	1.50	1.22	1.86	0.005	0.42	0.23	0.77
IL-6	< 0.001	1.06	1.04	1.09	0.024	1.06	1.01	1.12
hs-CRP	< 0.001	1.03	1.02	1.05	0.99	1.00	0.97	1.03
TG	< 0.001	1.02	1.01	1.03	0.24	0.98	0.95	1.01
LDL-C	< 0.001	1.04	1.03	1.06	0.40	0.98	0.95	1.02
iPTH	< 0.001	1.01	1.005	1.01	0.007	1.01	1.002	1.01
P	< 0.001	3.28	1.87	5.76	0.85	1.09	0.43	2.81
MK	< 0.001	1.001	1.00	1.001	0.001	1.001	1.00	1.002

IL-6, interleukin-6; hs-CRP, high sensitivity C-reactive protein; TG, total triglyceride; LDL-C, low density lipoprotein-cholesterol; iPTH, intact parathyroid hormone; P, phosphorus; MK, midkine; DM, diabetes mellitus; HTN, hypertension

**Table 6:** Predictors of carotid atherosclerosis using ROC curve analyses

Variables	Cut-off value	AUC	Sensitivity (%)	Specificity (%)	PPV (%)	NPV (%)	*P* value
Age (yrs)	>44	0.868	95	76.8	56.43	96.42	< 0.001
Creatinine (mg/dL)	>2.9	0.685	78.3	56.8	44.33	83.54	< 0.001
IL-6 (pg/mL)	>27.1	0.767	68.5	84.8	64.91	82.30	< 0.001
hs-CRP (mg/L)	>31	0.774	86.7	62.4	50.98	90.36	< 0.001
TG (mg/dL)	>178	0.690	63.3	84.8	57.57	81.51	< 0.001
LDL-C (mg/dL)	>116	0.748	76.7	71.2	53.48	85.85	< 0.001
iPTH (pg/mL)	>185	0.764	76.7	73.6	50	84.94	< 0.001
P (mg/dL)	>6.2	0.671	51.7	91.2	47.94	77.67	< 0.001
MK (pg/mL)	>2150	0.855	98.3	61.6	54.62	98.7	< 0.001

ROC, Receiver operating characteristics; AUC, area under the curve; PPV, positive predictive value; NPP, Negative predictive value; IL-6, interleukin-6; hs-CRP, high sensitivity C-reactive protein; TG, total triglyceride; LDL-C, low density lipoprotein-cholesterol; iPTH, intact parathyroid hormone; P, phosphorus; MK, midkine

### Demographic and laboratory characteristics of CAC groups

We then classified the CKD patients based on their CAC score into two groups; mild to moderate CAC group, in addition to severe CAC group. The median age of patients with mild to moderate CAC who included 73 males and 53 females was 43 years. Sever CAC patients who consisted of 30 males and 29 females had a median age of 43.5 years. Severe CAC patients were older than those with mild to moderate CAC (*p* < 0.001). No significant difference was found between the two groups regarding HTN, DM, smoking, and BMI. Sever CAC group had a considerably lower eGFR (*p* < 0.001) and significantly greater levels of serum creatinine (*p* < 0.001) and blood urea (*p* < 0.001) compared to mild to moderate CAC group. Severe CAC patients demonstrated significantly elevated serum levels of TNF-α, IL-6 and hs-CRP (*p* = 0.001, *p* = 0.002 and *p* = 0.003, respectively) in comparison to patients with mild to moderate CAC. Additionally, patients with severe CAC exhibited significantly higher serum levels of TC (*p* < 0.001) and LDL-C (*p* < 0.008), iPTH (*p* = 0.02) and phosphorus (*p* < 0.001) compared to patients with mild to moderate CAC. Furthermore, we found that severe CAC group had a considerably higher serum level of MK (*p* < 0.001) and a significantly increase of CIMT (*p* < 0.001) in comparison to mild to moderate CAC group (Table 7).

**Table 7:** Demographic and laboratory parameters of severe CAC and low to moderate CAC groups

Variables	Mild to moderate CAC group n = 126	Sever CAC group n = 59	*P* value
Age (yr)	43 (12)	43.5 (15.25)	< 0.001
Sex, *n* (%)			
Male	73 (57.9%)	30 (50.8%)	0.36
Female	53 (42.1%)	29 (49.2%)	
HTN, *n* (%)	40 (31.7%)	27 (45.8%)	0.06
DM, *n* (%)	42 (33.3%)	27 (45.8%0)	0.1
Smoking, *n* (%)	37 (29.4%)	22 (37.3%)	0.28
BMI (kg/m2)	22.4 (1.12)	22.97 (2.48)	0.11
Serum creatinine (mg/dL)	2.4 (1.45)	3.45 (1.3)	< 0.001
Blood urea (mg/dL)	62 (23)	74.5 (28.25)	< 0.001
eGFR (ml/min/1.73 m2)	31 (21.5)	18.5 (7.5)	< 0.001
Hb (gm/dL)	10 (1.5)	8.8 (2.3)	0.71
TNF-α (pg/mL)	14.6 (15.45)	36.25 (30.1))	0.001
IL-6 (pg/mL)	14.9 (10)	29.5 (44.85)	0.002
hs-CRP (mg/L)	30 (16)	48 (48)	0.003
TC (mg/dL)	180 (20)	210 (38)	< 0.001
TG (mg/dL)	160 (23)	220 (92.5)	0.12
LDL-C(mg/dL)	110 (24.5)	122 (37)	0.008
HDL-C (mg/dL)	35 (5)	35 (8.5)	0.29
iPTH (pg/mL)	168 (218)	338.5 (255)	0.02
P (mg/dL)	5.9 (0.7)	6.3 (0.88)	< 0.001
Total Ca (mg/dL)	7.9 (0.5)	7.8 (0.6)	0.79
CIMT (mm)	0.7 (0.3)	0.95 (0.8)	< 0.001
MK (pg/mL)	1460 (945)	3350 (2345)	< 0.001

Hb, hemoglobin; HTN, hypertension; DM, diabetes mellitus; BMI, body mass index; TNF-α, tumor necrosis factor-α; IL-6, interleukin-6; hs-CRP, high sensitivity C-reactive protein; TC, total cholesterol; TG, total triglyceride; HDL-C, high density lipoprotein-cholesterol; LDL-C, low density lipoprotein-cholesterol; iPTH, intact parathyroid hormone; P, phosphorus; Ca, calcium; CIMT, carotid intima media thickness; MK, midkine; CAC, coronary artery calcification, Continuous variables were expressed as median (interquartile range), while categorical variables were expressed as number and percentage

### Predictors of CAC in the CKD patients

Age (*p* < 0.001), serum creatinine (*p* < 0.001), DM (*p* = 0.02) and HTN (*p* = 0.049) were all independently associated with CAC, according to univariate regression. Additionally, hs-CRP, and IL-6 were found to be significantly associated with CAC (*p* = 0.003, and *p* < 0.001, respectively). Furthermore, it was found that MK (*p* < 0.001), TG (*p* = 0.002), LDL-C (*p* = 0.006), phosphorus (*p* = 0.003), and iPTH (*p* = 0.014) were all significant risk factors for the development of CAC (Table 8). Serum MK and serum creatinine were independent predictive risk factor of CAC, according to multivariate regression (*p* = 0.008, and *p* = 0.001, respectively) (Table 9). Furthermore, ROC curve revealed accuracy of MK in predicting CAC (AUC = 0.592, *p* < 0.037) (Fig. 4). ROC curve analyses revealed that age (AUC = 0.62, *p* = 0.006), iPTH (AUC = 0.658, *p* < 0.001), P (AUC = 0.631, *p* = 0.002), and MK (AUC = 0.592, *p* = 0.037) were consistently predictive of CAC. Furthermore, ROC curve found that IL-6 (AUC = 0.586, *P* = 0.063), hs-CRP (AUC = 0.578, *p* = 0.089), TG (AUC = 0.566, *p* = 0.138), LDL-C (AUC = 0.572, *p* = 0.109), and serum creatinine (AUC = 0.552, *p* = 0.256) weren’t predictive for CAC (Table 1^0^).

**Table 8:** Univariate linear regression to detect predictors of CAC in the studied CKD patients

Variables	Univariate linear regression						
	Unstandardized Coefficients		Standardized Coefficients	t	*P* value	CI	
	B	SE	Beta			Lower bound	Upper bound
Age	12.87	2.66	0.336	4.83	< 0.001	7.61	18.13
Smoking	88.39	46.53	0.139	1.90	0.06	−3.41	180.20
DM	108.67	44.57	0.177	2.44	0.02	20.74	196.59
HTN	89.36	45.08	0.145	1.98	0.049	0.41	178.31
Creatinine	96.12	12.01	0.509	8.01	< 0.001	72.42	119.79
IL-6	4.38	1.11	0.279	3.93	< 0.001	2.18	6.58
hs-CRP	2.76	0.93	0.215	2.97	0.003	0.93	4.60
TG	1.79	0.56	0.230	3.19	0.002	0.68	2.89
LDL-C	2.84	1.02	0.201	2.77	0.006	0.82	4.86
iPTH	0.45	0.18	0.180	2.48	0.014	0.09	0.81
P	99.33	32.69	0.219	3.04	0.003	34.83	163.83
MK	0.07	0.009	0.484	7.47	< 0.001	0.05	0.09

IL-6, interleukin-6; hs-CRP, high sensitivity C-reactive protein; TG, total triglyceride; LDL-C, low density lipoprotein-cholesterol; iPTH, intact parathyroid hormone; P, phosphorus; MK, midkine; SE, standard error; CI, confidence interval

**Table 9:** Multivariate linear regression to detect predictors of CAC in the studied CKD patients

Variables	Multivariate linear regression						
	Unstandardized Coefficients		Standardized Coefficients	T	*p* value	Collinearity statistics	
	B	SE	Beta			Tolerance	VIF
Age	−0.07	3.05	−0.002	−0.02	0.98	0.61	1.65
Smoking	20.79	46.39	0.033	0.45	0.65	0.72	1.38
DM	35.79	52.07	0.058	0.69	0.49	0.53	1.87
HTN	57.23	48.37	0.093	1.18	0.24	0.63	1.59
Creatinine	58.41	17.95	0.310	3.25	0.001	0.43	2.35
IL-6	−0.05	1.57	−0.003	−0.03	0.97	0.38	2.59
hs-CRP	0.47	1.08	0.037	0.44	0.66	0.54	1.84
TG	−1.34	0.89	−0.173	−1.50	0.13	0.29	3.44
LDL-C	−0.01	1.18	−0.001	−0.01	0.99	0.55	1.82
iPTH	0.18	0.19	0.074	0.99	0.32	0.69	1.43
P	17.19	33.39	0.038	0.51	0.61	0.71	1.41
MK	0.05	0.02	0.338	2.68	0.008	0.24	4.12

IL-6, interleukin-6; hs-CRP, high sensitivity C-reactive protein; TG, total triglyceride; LDL-C, low density lipoprotein-cholesterol; iPTH, intact parathyroid hormone; P, phosphorus; MK, midkine; SE, standard error; VIF, variance inflation factor

**Table 10:** ROC curve analyses to detect predictors of CAC in the studied patients

Variables	Cut-off value	AUC	Sensitivity (%)	Specificity (%)	PPV (%)	NPV (%)	*P* value
Age (yrs)	>41	0.621	77.5	46.4	75.3	45.1	0.006
Creatinine (mg/dL)	>2.9	0.552	58.9	55.4	64.55	35.44	0.256
IL-6 (pg/mL)	>21.6	0.586	44.4	73.6	87.87	35.96	0.063
hs-CRP (mg/L)	>27	0.578	70.5	46.4	74.59	39.68	0.089
TG (mg/dL)	>170	0.566	48.1	71.4	78.04	36.89	0.138
LDL-C (mg/dL)	>126	0.572	34.1	83.9	81.48	35.11	0.109
iPTH (pg/mL)	>285	0.658	48.8	80.4	84	40	< 0.001
P (mg/dL)	>6.1	0.631	47.3	78.6	81.81	38.88	0.002
MK (pg/mL)	>2650	0.592	45	78.6	81.69	37.71	0.037

ROC, Receiver operating characteristics; AUC, area under the curve; PPV, positive predictive value; NPP, Negative predictive value; IL-6, interleukin-6; hs-CRP, high sensitivity C-reactive protein; TG, total triglyceride; LDL-C, low density lipoprotein-cholesterol; iPTH, intact parathyroid hormone; P, phosphorus; MK, midkine

## Discussion

All patients in the CKD subgroups of our research exhibited statistically higher levels of MK as CKD worsened. Serum creatinine was found to be a significant independent factor impacting the serum MK levels. The human studies exploring the association between MK and CKD are very limited. Our study is in line with the findings of Campbell et al. who revealed that MK levels increased progressively with CKD stage. Additionally, they found that eGFR was an independently associated factor with MK level [18]. This is also similar to Fujisawa et al. who investigated serum MK levels in HD patients. The serum levels of HD patients and controls didn’t differ significantly prior to HD. However, during the first few HD sessions using heparin, serum MK levels surged dramatically and then gradually declined. Heparin administered intravenously caused a similar abrupt rise in MK in normal controls, although the subsequent decline was also rapid. In an in vitro research, MK was released from cultured blood vessels in a time- and heparin-dose-dependent manner, but not from the peripheral leukocytes. These findings suggest that in HD patients, MK is mostly released from endothelial cells as soon as heparin is administered, and that renal dysfunction causes MK to gradually disappear from the blood [43]. Our research contradicts the research by Wang et al. who showed that serum MK was not linked to changes in renal function or to hospitalisations or deaths related to CKD in older women with normal or slightly impaired renal functions, indicating that serum MK may not be a reliable biomarker for CKD progression [44]. Wang et al. hypothesized that the variation in study population characteristics was probably the cause of the disparity between their results and earlier findings. Unlike other cohorts with established renal disease, they investigated an elderly yet healthy cohort with few comorbidities and disease burden that was comparable to the general population [44].

These findings can be attributed to the possibility of increased intrinsic renal tubular production, altered renal clearance, and the pro-inflammatory nature of CKD. There is a possibility that CKD will result in either increased intrinsic or systemic renal production. This is consistent with histologically observed increased MK in the tubules of human diabetic kidney biopsies and animal studies demonstrating increased MK production in the renal tubules in streptozotocin-induced diabetic kidney disease [45]. Furthermore, this could be explained, for example, by the fact that the renal tissue remnants had relatively few functioning tubular cells that were able to produce MK. Serum MK levels in dialysis patients continued to surge in response to heparin, indicating that the MK bound in the glycocalyx proximal to the endothelium is still accessible and mobile [43]. MK has also been linked to inflammation [18]. Our research revealed that TNF-α, IL-6 and hs-CRP were found to be statistically significant independent factors affecting MK levels, according to univariate regression. MK is known for its cytokine-like characteristics. MK acts as an immunomodulatory agent through promoting the production of cytokines and chemokines as well as the migration and activation of immune cells to regions of inflammation or injury. Elevated serum MK is associated with autoimmune and inflammatory diseases such as different forms of CKD, rheumatoid arthritis, and atherosclerosis [45–48], indicating that the immunomodulatory effect of MK is likely to be deleterious under chronic conditions. The chemotactic characteristics of MK are shown to be mediated by heparin binding, as heparin removal inhibits MK-induced cellular migration [49].

It is not surprising that elevated serum MK is seen as proteinuria increases, but it is noteworthy that angiotensin blocking is linked to decreased serum MK levels. In CKD, proteinuria is linked to gradual and chronic tubulointerstitial damage. Several mechanisms have been found for this, but the processes are not well understood [18, 50]. The fibrotic and proinflammatory effects of a chronically increased intrarenal and/or urinary MK levels may be one mechanism by which proteinuria drives gradual progressive renal damage, with MK demonstrated to be upstream from the identified drivers of induced injury, such as chemokines like TGF-β and osteopontin [51]. In view of the findings by Hobo and colleagues, this association with angiotensin blockage (ACE-I/ARB) was very noteworthy [52]. They demonstrated that MK upregulates the renin-angiotensin system (RAS) and that inhibiting MK downregulates the RAS and lessens renal effects in an animal model of hypertensive 5/6 nephrectomy. RAS activation in CKD is known to contribute to CKD-associated hypertension, while blockage protects the kidneys by reducing proteinuria and GFR loss [50, 53]. Although the finding that angiotensin blockage is linked to decreased MK levels does not establish a causal relationship, it does suggest that MK may be a mechanism by which ACE-I or ARBs provide renal protection in CKD patients [18].

We discovered that CKD subgroups had statistically greater rates of carotid atherosclerosis than healthy participants. Furthermore, CKD stage-5 patients had significantly higher rates than CKD stage-3 patients. This is consistent with previous findings which discovered that non-HD CKD patients had greater rates of carotid atherosclerosis than those of the controls [54, 55]. Atherosclerosis in CKD can be caused by aberrant lipid modification, vascular calcification, uremic toxins, endothelial dysfunction, oxidative stress, and inflammation [56].

We found that MK was significantly associated with chronic inflammation and hyperlipidemia in CKD patients. Additionally, we revealed that serum MK was found to be a reliable predictor of carotid atherosclerosis in the CKD patients. There are no human studies exploring the association of MK with carotid atherosclerosis in the CKD patients. Few studies investigated the role of MK expression in atherosclerosis of the general population. Evaluation of atherosclerotic lesions from PAD patients revealed MK expression in the thickened intima of fatty streaks, especially in endothelial cells, inflammatory cells, and smooth muscle cells. Additionally, MK was found in advanced atherosclerotic lesions, primarily in the intimal cells and extracellular matrix [57]. Similarly, serum MK level was considerably higher in patients with severe PAD compared to healthy participants. It was surprising that serum MK showed no association with common risk factors as sex, age, obesity, HTN, DM, smoking or elevated cholesterol [19]. However, another research provided evidence linking elevated MK levels to atherosclerosis risk factors, including HTN, high LDL-C and TC, even though it didn’t specifically explore this relationship in patients with atherosclerotic CVD [58]. MK was also included as a biomarker in a clinical and biomarker score intended for predicting the existence of extensive CAD [59]. Surprisingly, neither myocardial ischemia nor necrosis changed MK levels in a recent study on MK levels in patients with CAD and acute ACS. Conversely, the use of heparin significantly increased the patients’ MK levels. The results of this study suggested that MK was probably not a significant biomarker for myocardial ischemia or necrosis [60].

MK is implicated in several phases of the atherosclerotic process, including macrophage lipid accumulation, inflammation, and neointima formation [17]. MK is particularly localised in the thickened intima of fatty streaks at the level of advanced atherosclerotic plaque. Here, it forms a dense and diverse cellular beach with pro-inflammatory properties alongside extracellular cells, vascular smooth muscle cells, and inflammatory agents, ensuring the advancement of the atherosclerotic process [17]. MK may be regarded as a dyslipidemia promoter since it increases the accumulation of lipids in macrophages. There is a directly proportionate association between MK and serum levels of LDL-C or TC, regardless of the presence of HTN [58]. Additionally, MK is crucial for the process of inflammation in the atherosclerotic plaques, as it increases the mRNA levels of pro-inflammatory cytokines like interferon-γ and interleukin-1β [61] and stimulates activation of T cell and differentiation of Th1 cell [62]. Additionally, MK stimulates monocyte adhesion to the arterial walls in order to facilitate their eventual differentiation into macrophages. Takemoto et al. showed in an in vitro investigation that administering MK is linked to elevated serum levels and controls monocyte chemoattractant protein-1 in aortic tissues, which causes macrophages to accumulate in atherosclerotic lesions [63]. MK exerts its atherogenic action by the suppression of macrophage apoptosis, which limits the advancement of atherosclerotic processes [64]. Suppression of MK expression or ablation of MK function decreases inflammation and activation of the immune response and improves the disease outcomes in a variety of long-term autoimmune/inflammatory diseases, like rheumatoid arthritis, atherosclerosis, experimental autoimmune encephalomyelitis (EAE) and different forms of CKD [45–47].

Our research findings showed that CAC was common in CKD patients and increased as the disease progressed. These findings were in line with previous investigations [54, 65, 66]. In our study, we reported a strong association between VC and hyperlipidemia, serum creatinine, chronic inflammation, and secondary hyperparathyroidism in CKD patients. CKD exhibited hypocalcaemia and hyperphosphatemia, which can result in secondary hyperparathyroidism and vitamin D deficiency. Both of these disorders are closely related to VC. Elevated levels of glucose, lipids, and low-density lipoproteins that are present in the endothelial lining [67], chronic inflammation, oxidative stress, the buildup of uremic toxins, the downregulation of calcification-inhibiting factors and the upregulation of calcification-promoting factors are additional risk factors of VC in CKD [68].

Our data found that serum MK was notably increased in severe CAC compared to mild to moderate CAC. We also reported that there was a significant association between MK and VC. Studies investigating MK role in VC haven’t been conducted in the CKD patients. Our study investigated the association of MK with VC in CKD for the first time. Researches demonstrating MK role in renin-angiotensin system (RAS) activation and cardiac hypertrophy during renal impairment offer convincing evidence that MK may act as a cytokine in CKD and aggravate the harmful vascular and hormonal responses that occur during the renal-cardiovascular syndrome [16, 69]. Considering its established atherosclerotic effects, MK may induce VC by promoting endothelial dysfunction, inflammation, and endothelial to mesenchymal transition (EndMT) of the vasculature, processes that have been shown to induce osteogenic de-differentiation of smooth muscle and endothelial cells [70–72]. Additionally, MK can lead to elevated circulating phosphate levels, which in turn stimulate the osteoblastic transition of vascular smooth muscle cells (VSMCs) by suppressing skeletal remodeling and stimulating bone resorption. Finally, given the evidence supporting MK role as a Wnt inhibitor, MK may serve as a circulating factor that directly controls the osteogenic transition process of VC in patients with CKD [73, 74].

In addition to the importance of serum MK as a biomarker, the involvement of MK in a wide range of diseases, such as CKD, malignancies, and inflammatory diseases of peripheral and central nervous system, suggests that it could be a promising therapeutic target. MK blockade is a potential therapeutic treatment, depending on the nature of the disease and the role of the growth factor within a pathological condition. MK stimulates tumor development, differentiation, and resistance to treatment in neoplastic disorders. Accordingly, MK-targeted approaches may have significant therapeutic promise, especially in malignancies where treatment resistance exists. Recent researches show that tumor resistance can be restored by blocking MK signaling in a variety of approaches. The strategies utilized to inhibit growth of tumor and restore tumor apoptosis in mice include the use of the small molecule inhibitor iMK [75], small interfering RNAs (siRNAs) [76], or MK blockage using anti-MK monoclonal antibodies [77]. Monoclonal antibodies directed against MK may allow targeting these tumorigenic effects in the CNS, however, current candidates still lack the necessary efficacy [78]. Anti-MK aptamers have also demonstrated therapeutic usefulness in treating autoimmune CNS disorders including multiple sclerosis [47].

Collectively, MK blockade harbors great promise as a therapeutic approach for malignant and autoimmune disorders of the peripheral and the central nervous systems. It is crucial to investigate whether iMK and anti-midkine monoclonal antibody can inhibit the progression of CKD and atherosclerosis. They may be further developed as therapeutic agents for CKD progression and atherosclerotic cardiovascular disease. Understanding these questions will provide insightful knowledge about the underlying mechanisms and accelerate the development of MK-targeted therapy.

The availability of two indicators of subclinical CVD and a well-characterized cohort of CKD patients without prevalent CVD are two of the study’s strengths. Despite searching through the main medical databases such as Google Scholar, PubMed, Scopus, and Web of Science, comprehensive researches exploring the relationship of serum MK with subclinical CVDs in non-dialysis CKD have not yet been conducted. Our research provides a pioneering investigation into measurement of serum MK and determining its association with subclinical CVDs in non-dialysis CKD patients. There are certain limitations to our research. Hemodialysis may have an impact on a patient’s MK level. Serum MK levels were measured only once, therefore it is impossible to know if they varied during the duration of the research. CIMT was assessed just once because of the study design, making it impossible to identify changes over time.

## Conclusion

All patients in the CKD subgroups exhibited statistically higher levels of MK as CKD worsened. CAC and carotid atherosclerosis were common in CKD patients and increased as the disease progressed. Using multivariate regression, carotid atherosclerosis was reliably predicted by MK, old age, serum creatinine, IL-6 and iPTH. Multivariate regression reported that CAC was consistently predicted by MK and serum creatinine. Multivariate regression and ROC curve revealed that MK was a reliable biomarker for carotid atherosclerosis and CAC. The relationship between MK and carotid atherosclerosis and CAC provides evidence to the concept that MK may have a role in the development of these conditions. Thus, we suggest that serum MK may act as a reliable biomarker for these subclinical CVDs in non-dialysis CKD patients. Furthermore, we can use it as an early marker of atherosclerotic burden because prompt early diagnosis and treatment could prevent cardiovascular morbidity and death in the future.

## Data Availability

The corresponding author can provide the datasets used and/or analyzed during the current research upon reasonable request due to ethical restrictions and privacy.
